# Assessment of supervised classifiers for the task of detecting messages with suicidal ideation

**DOI:** 10.1016/j.heliyon.2020.e04412

**Published:** 2020-08-03

**Authors:** Roberto Wellington Acuña Caicedo, José Manuel Gómez Soriano, Héctor Andrés Melgar Sasieta

**Affiliations:** a Carrera de Tecnología de la Información, Universidad Estatal del Sur de Manabí, Ecuador; b iLife Company, Spain; c Departamento de Ingeniería, Sección de Ingeniería Informática, Escuela de Posgrado, Pontificia Universidad Católica del Perú, Lima, Peru

**Keywords:** Computer science, Suicidal ideation, Supervised classifiers, Machine learning, Social networks, Automatic classification, Suicide

## Abstract

According to the World Health Organization (WHO) close to 800,000 people worldwide die by suicide each year, and many more attempts to do it. In consequence, the WHO recognizes suicide as a global public health priority, which affects not only rich countries but poor and middle-income countries as well. This study makes a systematic analysis of 28 supervised classifiers using different features of the corpus Life to detect messages with suicidal ideation and depression to know if these can be used in an automatic prevention online system.

The Life Corpus, used in this research, is a bilingual text corpus (English and Spanish) oriented to the detection of suicide ideation. This corpus was constructed retrieving texts from several social networks and its quality was measured using mutual annotation agreement. The different experiments determined that the classifier with the best performance was KStar, with the corpus features POS-SYNSETS-NUM, achieving the best results with the ROC Area metrics of 0,81036 and F-measure of 0,7148. The present research fulfilled the objective of discovering which supervised classifiers and which features are the most suitable for the automatic classification of messages with suicidal ideation using the Life Corpus.

Also, given the imbalance of the results, a new precision measure was developed called the Two-dimensional Accuracy and Recovery Index (GDP), which can provide better results, in unbalanced systems, than the usual measures to assess the quality of the results (measure F, Area ROC), and thus increase the number of messages at risk of suicidal ideation, detected at the cost of receiving more messages that are not related to suicide or vice versa.

## 1 Introduction

The world population, according to a statistical collection of the web site We Are Social [48], was, as of January 2019, more than 7.676 billion people. Of this population more than 4.388 billion are internet users, 3.434 billion are asset users of social media, 5.112 billion are cell phone users and 3.256 billion are users of mobile social media. As social media users represent a vast portion of the world population, they are a cohort subject to frequent analysis in the field of opinion mining, based on the content they publish on social networks.

Furthermore, according to the World Health Organization (WHO) close to 800,000 people in the world death by suicidal each year, while many more attempts to do it [52]. In consequence, the WHO recognizes suicide as a global public health priority, which affects not only rich countries but poor and middle-income countries as well [51]. Suicide is a serious public health problem, affecting all age groups, and in 2015 was the second leading cause of death in the world for the age group of 15–44 years, and while in Europe, suicide has become the leading cause of violent death [15].

However, suicide is a problem that is preventable through timely interventions when these are based on trustworthy and often inexpensive data. At the same time, efficient solutions require a multi-sector and integrated strategy of suicide prevention [32]. In the report, “Suicidal prevention: A global imperative”, in the Mental Health Action Plan 2013–2020, published in 2014 [32], the WHO highlights how each suicide is a tragedy that affects families, communities, and countries, and has a lasting effect on the relatives of the victim. Faced with these realities, the member states of the WHO have committed to reducing national rates of suicide by 10% before the year 2020, to accomplish this they agreed to elaborate and put into practice comprehensive national strategies of suicide prevention, to improve their information systems, update their scientific data, and perform collaborative university research on mental health [32].

Before taking the fateful decision to end their own lives, suicide victims generally pass through a period of deep personal suffering, frequently endured in silence, and therefore to predict whether someone is going to commit suicide has been an all but impossible task [14, 20, 23, 24, 36]. It is nonetheless possible to detect factors that contribute to the risk of suicide, through standard clinical tools operated by well-trained doctors [4, 5, 39].

In this framework, the science offers the opportunity to understand the biological markers and psychological markers, related to suicide, while computer science, specifically Natural Language Processing, offers the opportunity of understanding the indicators of suicidal thoughts [25, 39] when these are expressed in written and spoken forms.

In fact, in 2008 Pestian and Matykiewicz [40] showed that the algorithms of automatic learning could better distinguish between, notes written by people who subsequently died by suicide and simulated suicide notes, compared to mental health professionals by a score of 71% vs 70%.

Furthermore, social networks have provided researchers with new platforms to deploy automated methods for the analysis of language to better understand the thoughts, feelings, beliefs, and personalities of each person [44], even mental illnesses like depression or Post-Traumatic Stress Disorder [42]. For example, some projects have used data from microblogs to build automatic learning models to identify suicidal bloggers at a level of precision of approximately 90% [55].

In this work, a series of systematic experiments have been carried out with all possible combinations of textual feature from the Life Corpus, and machine learning algorithms. The main objective is to find out what features of the corpus and which classification algorithms are the most suitable to carry out the detection of messages with suicidal ideation and depression. As we can see in Section 2, a whole range of different approaches to the problem are available. However, these approaches have been developed using corpus without quality measures nor suitable to solve this task. The contribution of this research to the state of art is an exhaustive analysis of different techniques and features for text classification applied to the suicide ideation detection using a scientifically validated corpus. Furthermore, this corpus was specifically built to be used for supervised machine learning approaches of messages which express suicide ideation [9]. Another advantage of this corpus is that it is available with a Creative Commons license.

Therefore, this document is organized as follows: Section 2 describes a review of researches on the use of technology for suicide prevention or suicide ideation detection. Next, Section 3 explains the methodology and resources used for developing this work. Later, in Section 4 the results are presented for discussion in Section 5, Section 6 Limitation. Finally, in the Section 7 conclusions are made, and future works are proposed.

## 2 Background

The task of detecting suicidal users through new technologies is a wide field covering different areas of knowledge, from the recognition of facial expressions to the preprocessing of human language, including the monitoring of geolocation or distinguishing the featured speech of people with depression [15].

Specifically, research on automatic systems for the detection of suicidal tendencies in written messages started in 1959, with the first Corpus of Original Notes (GSN), created to identify the textual characteristics of the suicide notes. It contained a sample of 66 suicide notes, of which half were genuine and the other half simulated [33]. In the same way, other more recently researchers also focused on distinguishing between real or simulated suicide notes [11, 25, 26, 34, 38, 39, 43, 46]. However, these works are not suitable for the detection of suicide messages, but only for the differentiation between real suicide notes and false ones, because this corpus do not have either example of non-suicide messages, or other types of messages than suicide notes.

The literature review shows that other research groups have focused on research works that use natural language processing to identify suicidal ideation and attempts in clinical databases [2, 3, 6, 13]. Other works are only based on statistical analysis, and they don't use natural language processing to identify suicide ideation and suicide attempts in its corpus [21]. Fernandes et al. [13] try to detect suicide ideation from texts written by medical staff, not by their patients. Therefore, they cannot be used in systems that aim to analyze messages written directly by people with mental illness in social networks.

Likewise, Naderi et al. [30] used natural language processing for other specific, but related, purposes: the quantification of the spread of anxiety and mental disorders on social networks. However, although it is a similar task, this work was focused on how this kind of messages with anxiety or mental disorders are spread on social networks, and not in its detection.

On the other hand, research works have been developed for the detecting of suicidal ideation, both with supervised and unsupervised methodologies, from twitter messages [21, 29, 31, 41, 53]; from Weibo [19, 54]; from Netlog [10]; and other microblogs [17]. The problem with this research is that the only ones that used a methodology and evaluate the quality of the corpus using metrics such as Cohen's Coefficient were Desmet [10], ODea [31] and Mowery [29]. Therefore, without knowing the degree of quality of the corpus, it cannot be deduced that the results of these works correspond to the solution of these systems in a real environment since said corpus could have some undetected bias when not using any scientific methodology in its construction.

Desmet et al [10] worked with posts in a German-language email forum in the social network Netlog, and used genetic algorithms to optimize the model through the selection of features and hyperparameters, such as bags-of-words, polarity lexicons, specific domain lexicons, typical models, surface features and entities names. The categories used were ``*Relevant*'', ``*Severe*'' and ``*Irrelevant*''. For text classification, they used Support Vector Machine and Naive Bayes, reaching 93% of F-measure for relevant messages and 70% for severe messages. This research demonstrated that with a big enough corpus, the detection of messages with suicidal ideation thoughts could be very effective.

ODea et al [31] focused on examining whether the level of concern for a suicide-related post on Twitter, could serve, to generate a training corpus for automatic learning models. The data collection obtained from Twitter consisted of 14,701 suicide-related tweets: 14% classified as ``strongly concerning'', 56% ``possibly concern'', and 29% as ``safe to ignore''. The metrics used were Recall and Precision. The classifiers used were Support Vector Machine and the Logistic Regression method. The algorithm with the best performance was Support Vector Machine with TF/IDF without filter, with 67% effectiveness.

Mowery et al [29] developed a comprehensive annotation scheme for manually annotating Twitter data with the diagnostic and statistical manual of mental disorders, edition DSM-5 [1], based on analyzing depression-related Twitter data. They tagged 9300 tweets and they found that 72.09% (6829/9473) of tweets containing relevant keywords were not indicative of depressive symptoms. The most prevalent symptoms were depressed mood and fatigue or loss of energy. Less than 2% of tweets contained more than one depression-related category.

Although ODea and Mowery [29, 31] developed corpora with a correct methodology, and quality measured, they have the disadvantage that it focuses only on a microblog (Twitter) whose writing, given its length, is very different from other networks. On the other hand, Desmet [10] only worked with the German language and for a specific social network.

To solve these issues, we decided to use the corpus Life [9], since apart from complying with the quality criteria we were looking for, their texts come from different sources. If we want to create a system that tracks the Internet, we cannot train that system with a single type of social network and less if it has certain peculiarities that make it very different. This corpus is oriented to detect messages with suicidal ideation and depression, in contrast to the others cited, is bilingual (English and Spanish) and does not focus on a single social network but instead combines texts from different sources: social networks, blogs, and forums both in the deep and the shallow web. Therefore, it is not orientated to a single data source as is the case for the other corpora mentioned in this investigation.

In our work, some of the machine learning techniques evaluated in the bibliography were used but with three advantages: 1) A systematic analysis was made of a large number of classifiers and not just a few; 2) We proceeded to try out with distinct features of corpus, each one of the classifier used; 3) Corpus Life was used [9], which has proven its quality through different metrics which will allow future comparisons with other systems or techniques; and 4) the use of standard metrics to evaluate the results, of F-measure, ROC Area, precision and recall in order to be comparable with the results of other works. The access to the Life Corpus is free under a Creative Commons license in https://github.com/PlataformaLifeUA, so that the experiments carried out in this research can be replicated.

Finally, also made sure that the results were statistically significant through an ANOVA analysis of the data. Despite these advantages, Life Corpus has the disadvantage that it is quite small due to its exhaustive quality of the build process.

## 3 Methodology

We used machine learning techniques to carry out a systematic analysis of all possible combinations of textual feature from the Life Corpus with 28 supervised classifier algorithms with Weka default parameters. The corpus, used in this research, is a bilingual corpus (English and Spanish) oriented to suicide, built upon texts from several social networks. The corpus quality was measured using a mutual annotation agreement (Cohen's Kappa Micro) obtaining a moderate agreement of 0.52. Finally, the significance of the results was measured statistically by means of variance analysis with one factor (ANOVA).

### 3.1 Life Corpus

For the present research, which focused on a supervised learning approach, we used the Life Corpus [27] developed by the research group from Natural Language Processing and Information Systems, ascribed to the University of Alicante, in the project *Sentiment Analysis Applied to Suicide Prevention in social networks* (ASAPS) [16].

The platform Life is a research framework whose main aim is to obtain the necessary resources for automatic detection of suicidal ideation, suicidal incitement, or depression symptoms, from written texts in social networks.

The corpus is composed of 102 texts in two languages: English (71 texts) and Spanish (31 texts), that are used together; collected from social networks such as Facebook, Twitter, Instagram, blogs and forums. The corpus has 2 different and independent classifications: Alert Level and Message Type. The first contains 4 different levels of alert which are the following:

**Immediate**: This is the highest alert level. Messages included here need an immediate referral to health care or emergency services. They clearly express the idea of being suicidal. Suicidal groups and instigator profiles are suitable here. Description of self-harming behavior with certain linguistic expressions such as “Today is the day.”
**Urgent**: The suicidal thought is noticeable but not immediate. Repetitive depressive thoughts are related to this category. Although methods of suicide are present, the text shows suitable relationships with its immediate environment. Consequently, phrases such as “I feel that I'm not anyone” and a description of self-harming behaviors are included.
**Possible**: Not every message has the same risk. Therefore, for this type of alert a possible message indicates a temporal episode of depression, sadness, dissatisfaction with life, etc. is included in this type of alert. ``Possible'' is related to cases in which there are doubts about whether the risk is present or not.
**No Risk**: This type includes texts in which suicide is not commented on in any way. Also, there are positive thoughts and encouraging messages, too.

The second classification defines the type of message in the following categories: Undefined, Auto-Pro-Suicide, Sadness/melancholy, Auto-No-Pro-Suicide, Depression, Irony, Citation, Mysticism, and Instigator.

Due to the reduced size of the corpus, we decided to use only the classification of Alert Level since this reduces the number of classes and we have more samples per class in order to achieve more statistical significance. Although the principal advantage of the corpus is that the quality has been assessed, there is the drawback that its classes are not balanced – as shown in Table 1.

**Table 1:** Number of samples for each “Alert Level” type.

Alert Level	Quantity	EN	ES
No risk	70 (68.6%)	45 (63.4%)	25 (80.6%)
Urgent	19 (18.6%)	15 (21.1%)	4 (12.9%)
Possible	8 (7.8%)	6 (8.5%)	2 (6.5%)
Immediate	5 (4.9%)	5 (7%)	0 (0%)

As we see in section 4, this imbalance in the corpus and the limited samples in each class indicate that the categories relating to some kind of risk caused us problems in the evaluation of the classifiers. Thus, we also decided to group the four original categories under in only two: Risk and No Risk. The Risk categories will contain all the alert messages which indicate some kind of risk (Urgent, Possible, and Immediate), totaling 32 examples, and the No-Risk categories will contain the remaining 70 examples. It should be noted that this corpus has been previously used in others research [37].

### 3.2 Feature extraction

The Life Corpus was preprocessed to extract different types of features, using Natural Language Processing techniques, and trying these out in different combinations to discover which features were better suited for the automatic classification of messages with suicidal ideation. The features used were:

**Bag of Words (WORD)** Bag of words of the document without any modification.
**Bag of Stems (STEM)** The words of the document though carrying out a process of stemming to reduce the variety of derivations of a single word.
**Bag of Lemmas (LEMMA)** Instead of using each word, its lemma was used with the same objective as the use of stem as well as to find out which technique (stemming or lemmatization) was better.
**Bag of SYNSETS (SYNSET)** The WordNet SYNSET of each term used in the corpus was obtained to expand the coverage of the corpus by choosing words with the same meaning instead of the literal word. These features were extracted using Freeling 4.0 [35].
**Bag of POS (POS)** The Part-Of Speech of each term is used as a feature.

The number of features for each type is shown in Table 2. With these features, an experiment was launched with each combination, for instance, bag of words, bag of words and POS, bag of words, POS and SYNSETS, and so on. Independently of the combination used, we repeated the experiment with and without stopwords and, also, keeping the numeric values or replacing them by a unique tag _NUM_. These two techniques were used to see if by reducing the number of features of the corpus we could get the same or better results.

**Table 2:** Number of features for each type of features, with and without stop-words.

	With numbers		Without numbers	
	With stopwords	Without stopwords	With stopwords	Without stopwords
WORD	1713	1474	1705	1466
STEM	1434	1261	1433	1254
LEMMA	1430	1260	1422	1252
LEMMA	1430	1260	1422	1252
POS	173	148	173	148

Immediately, experiments with different combinations of features were run for each classifier: words, words and lemmas, words and lemmas and POS, only lemmas, only POS, POS and SYNSETS, and so on. For each of these combinations, there were experiments with stopwords and others without them or with the substitute number label.

### 3.3 Machine learning approaches

In this section, we offer a general description of the methodology used to determine the supervised classifier and the most appropriate characteristics for the automatic classification of messages with suicidal ideation using the Life Corpus.

The experiments were conducted by extracting certain features of the Life as explained in section 3.2, using 124 different combinations. That to say, with the features extracted from the corpus: WORD, STEM, LEMMA, SYNSET, and POS, experiments were carried out for each of these features individually.

To perform the experimentation, we used Weka software [18, 49] and the parameters that were chosen for each one of the algorithms were those that come by default. For every single one of the combinations described, a model was trained with each one of the 28 classifier algorithms that appear in Table 3 along with the parameters used, in order to facilitate the reproduction of these. At the end, 3,472 experiments were made, combining the features with each one of the classifier algorithms (124 feature combinations and 28 classifiers).

**Table 3:** Algorithms and default parameters used in the experiments.

Classifier algorithm	Algorithm parameters used
BayesNet	-D -Q weka.classifiers.bayes.net.search.local.K2 -- -P 1 -S BAYES -E weka.classifiers.bayes.net.estimate.SimpleEstimator -- -A 0.5
SimpleLogistic	-I 0 -M 500 -H 50 -W 0.0
SMO	-C 1.0 -L 0.001 -P 1.0E-12 -N 0 -V -1 -W 1 -K "weka.classifiers.functions.supportVector.PolyKernel -C 250007 -E 1.0″ IBK
IBK	-K 1 -W 0 -A “weka.core.neighboursearch.LinearNNSearch -A ∖“weka.core.EuclideanDistance -R first-last∖”” KSTAR
Kstar	-B 20 -M a
AdaBostM1	-P 100 -S 1 -I 10 -W weka.classifiers.trees.DecisionStump ATTCLASS
Baggind	-P 100 -S 1 -num-slots 1 -I 10 -W weka.classifiers.trees.REPTree -- -M 2 -V 0.001 -N 3 -S 1 -L -1 -I 0.0
CVParamSelection	-X 10 -S 1 -W weka.classifiers.rules.ZeroR
MultiClassifier	-M 0 -R 2.0 -S 1 -W weka.classifiers.functions.Logistic -- -R 1.0E-8 -M -1 -num-decimal-places 4
MultiClassUp	sin parámetros
MultiSchema	-X 0 -S 1 -B weka.classifiers.rules.ZeroR
RamdomCommittee	-S 1 -num-slots 1 -I 10 -W weka.classifiers.trees.RandomTree -- -K 0 -M 1.0 -V 0.001 -S 1
RandomFiltClass	-S 1 -F weka.filters.unsupervised.attribute.RandomProjection -W weka.classifiers.lazy.IBk -- -K 1 -W 0 -A weka.core.neighboursearch.LinearNNSearch RANDOMSUB
RandomSubSpace	-P 0.5 -S 1 -num-slots 1 -I 10 -W weka.classifiers.trees.REPTree -- -M 2 -V 0.001 -N 3 -S 1 -L -1 -I 0.0 STACKING
Stacking	-X 10 -M weka.classifiers.rules.ZeroR -S 1 -num-slots 1 -B weka.classifiers.rules.ZeroR VOTE
Vote	-S 1 -B weka.classifiers.rules.ZeroR -R AVG
WeighteDistances	-S 1 -W weka.classifiers.rules.ZeroR
InputMappedClassifier	-I -trim -W weka.classifiers.rules.ZeroR
DecisionTable	-X 1 -S weka.attributeSelection.BestFirst
Jrip	-F 3 -N 2.0 -O 2 -S 1
OneR	-B 6
PART	-M 2 -C 0.25 -Q 1
ZeroR	-output-debug-info
HoeffdingTree	-L 2 -S 1 -E 1.0E-7 -H 0.05 -M 0.01 -G 200.0 -N 0.0
J48	-R -N 3 -Q 1 -M 2
LMT	I -1 -M 15 -W 0.0
RandomForest	-K 0 -M 1.0 -V 0.001 -S 1
RamdomTreede	M 2 -V 0.001 -N 3 -S 1 -L -1 -I 0.0

To assess the statistical significance of the results, we repeated each experiment with 30 different random divisions of the corpus to perform an ANOVA statistical test. In these experiments, we used the values given by Weka software-defined by Equations: F-Measure (Eq. 1), ROC Area (Eq. 2), Precision (Eq. 3) and Recall (Eq. 4).

(1) $$F_{1}=2\cdot \frac{precision\cdot recall}{precision+recall}$$

(2) $$ROCArea:TPRate=\cdot \frac{TP}{TP+FN},FPRate=\cdot \frac{FP}{TN+FP}$$

(3) $$precision=\frac{TP}{TP+FP}$$

(4) $$recall=\frac{TP}{TP+FN}$$

Although these measures are the most used in evaluating classifiers, we have some issues recognising the most suitable model for suicide detection tasks.

The final objective of these classifications is to detect the highest quantity cases of suicidal ideation, trying to minimize the false positives so as not to overload the emergency services with false alarms, but maximizing the true positives of the class that suppose some risk. To our knowledge, no metric reflects this value, nor do we believe that there is a suitable value, it rather depends on the preferences of the emergency service workers or on how the messages are prioritized. For example, if in a suicidal prevention service there are generally few alerts, it might be interesting to expand the coverage until too many alerts are generated, or instead, we may be interested in making sure that the messages that arrive are mostly true positives, thus reducing false alarms [8]. At both ends, the classifier algorithms or the used features would be different, and, initially, we could not discard any. For this reason why we have decided to represent the classifiers with two-dimensional metrics, where the x-axis represents the proportion of hits in the classes that represent some level of risk divided by the total risk samples, as it appears in Eq. (5), and on the y-axis, the proportion of hits in the classes that represent little or no risk compared to the total as shown in Eq. (6).

(5) $$precision_{risk}=\frac{TP_{risk}}{TP_{risk}+FP_{risk}}$$

(6) $$precision_{no\_risk}=\frac{TP_{no\_risk}}{TP_{no\_risk}+FP_{no\_risk}}$$

In this way, we represent in a single graph the results of the classifiers in which the points which interested us most in the first instance will be those that are represented closest to the value 1 of these two metrics. However, and as we will see, the more we try to increase the first value the more it will tend to reduce the second and vice versa. Thus, depending on our needs, we will have to choose the algorithms that most interest us, as we will see in results section.

Once the results of the experiments with Weka were obtained, they were processed through the Chi^2^ statistic to verify which were statistically normal and which were not. Eq. (7).

(7) $$X^{2}=\sum_{i=1}^{k}{\left(\frac{fo_{i}-fe_{i}}{fe_{i}}\right)}^{2}>X^{2}\;The\;hypothesis\;is\;rejected$$

where *fo*_*i*_ is the observed frequency and *fe*_*i*_ is the expected frequency.

In addition to discriminating the experiments with statistically abnormal values, the Chi^2^ statistic allowed to discriminate those experiments in which the standard deviation is equal to zero (which were the classifiers that, regardless of the sample of entries, always classified it as “No-Risk “, that is, of the class with the most samples). As a result, it emerged that 1,216 experiments were viable according to the proposed formula these were the ones we considered to continue the process.

Immediately the ANOVA statistic was executed to compare similarities and statistical differences among the 1,216 experiments obtained from the statistical process Chi^2^. (8), (9), (10), (11)

(8) $$SS_{TOTAL}=\sum {X_{i}}^{2}-\frac{T^{2}}{N}\;\;T=Total\;sum\;of\;times$$

(9) $$SS_{TRAT}=\frac{1}{30}\sum {T_{i}}^{2}-\frac{T^{2}}{N}\;\;T_{i}=Sum\;by\;treatment$$

(10) $$SS_{ERROR}=SS_{TOTAL}-SS_{TRAT}\;\;$$

(11) $$F=\frac{\frac{SS_{TRAT}}{a-1}}{\frac{SS_{ERROR}}{N-a}}\;\;$$

a: Number of classifiers N: Number of times

As a result of executing a comparison of all against all, among the statistically significant experiments, using the ANOVA statistic, we obtained 739,935 comparisons, of which 71,983 were statistically significant.

## 4 Results

To obtain the results, we have run 3,472 experiments with different combinations of features and classifier algorithms, as described in Section 3.3. We have used the Life Corpus, with the original 4 classes (``No-Risk'', ``Possible'', ``Urgent'', and ``Immediate''), after which we grouped the characteristics into only two (``No-Risk'' and ``Risk''), where the 2 classes that indicate some risk of suicidal ideation or depression have been consolidated into one. We have carried out a statistical analysis of the results using an ANOVA with a 95% confidence margin. In Table 4 the 20 best results of the F-measure and ROC Area are shown for the Life Corpus version with four classes.

**Table 4:** Results of the first values of the evaluation of the corpus for differentfeatures and different classifiers for the version of the corpus with 4 classes.

Corpus features	Classifier	F1	ROC Area
(1) POS, NUM | (2) POS,SYNSET,NUM	KStar	0.715	0.749
(3) POS, SYNSET | (4) POS	Kstar	0.715	0.749
(5) POS,SYNSET, NUM | (6) POS,SYNSET	RandomCommittee	0.704	0.718
(7) POS,STEM, SYNSET,LEMMA, WORD,NUM	RandomCommittee	0.703	0.718
(8) STOPWORD, SYNSETS,LEMMA	RandomCommittee	0.702	0.718
(9) POS,STEM, WORD,NUM	RandomCommittee	0.701	0.718
(10) STEM, SYNSETS | (11) STEM	Kstar	0.700	0.724
(12) STOPWORD, STEM,SYNSETS, LEMMA,WORD	Kstar	0.700	0.724
(13) WORD, NUM | (14) STOPWORD, LEMMA,WORD	Kstar	0.700	0.724
(15) STOPWORDS, STEM,LEMMA, WORD	Kstar	0.700	0.724
(16) STOPWORDS, STEM,LEMMA, WORD,NUM	RandomCommittee	0.699	0.704
(17) STOPWORDS, STEM,SYNSETS, LEMMA	RandomCommittee	0.699	0.704
(18) WORD | (19) POS,STEM, LEMMA,WORD	RandomCommittee	0.699	0.704
(20) LEMMA, WORD,NUM	SMO	0.699	∗

POS: Part of Speech; SYNSET: Wordnet Synsets; WORD: Bag of Words; STEM: Stemof words; LEMMA: Lemma of words; NUM: without numbers; STOPWORD: withoutstopwords.

Note:each combination of training features is numered by (#), (∗) This value is notstatistically significant.

According to the results presented in Table 4, we found that the classifier KStar was the best performer across the experiments, followed by the classifiers RandomCommittee and SMO. It was also observed that the machine learning approach with the best performance was the KStar classifier which achieved a greater reduction of features: POS (Part of Speech), NUM (substitution of the different numbers by a single keyboard NUM and the SYNSET of WordNet that represents the semantic category of the term).

However, we perceived that the POS with different terms of the texts the same results were achieved and that with the combination with other features they became more complex. This seems to indicate that, for a corpus on suicidal ideation of this size, the lexical category is more than enough to get the best results. This agrees with previous work shown in the bibliography described in the Section 2 where it was indicated that our state of mind can manifest itself in the linguistic features we use to communicate [43].

Another classifier that gave similar but significantly lower results was the RandomComittee. In this one we found the same pattern, where POS was the predominant feature in combination with others, there being nothing statistically significant to differentiate between them. Minimal differences were found with the features of STEM and WORD, which represented bags of words, the first with stemmer and the second without, whether we eliminated the stopwords or not, or when we added new features such as the SYNSET or the lemma of the words. The other combinations of features and classifiers produced significantly lower values.

Due to the small size of the corpus, there were classes in which the number of samples was very low, and it was difficult to create a learning model that was capable of classifying the samples of that class correctly. This could cause insignificant results for those classes not to be significant and to generate noise in the evaluation. That is why we decided to merge the 3 risk classes (Possible, Urgent, and Immediate) into one. In this way, we arrived at a corpus with two classes: The first messages without any risk of suicide with 70 samples (No risk) and the second with messages with some level of risk with 32 texts (Risk). The results of this evaluation can be seen in Table 5.

**Table 5:** Results of the first values of the evaluation with different classifier and different categories for the version of the corpus with 2 classes (“Risk” and “NoRisk”).

Corpus features	Classifier	F1	ROC Area
(1) POS,SYNSETS,NUM	KStar	0.746	0.810
(2) POS,SYNSETS, lemma,word	RandomCommitte	0.716	0.768
(3) stopwords,POS,stem,NUM	RandomizableFiltered	0.715	0.675
(4) stopwords,POS,stem, SYNSETS,lemma, word,NUM	RandomizableFiltered	0.713	0.671
(5) POS,NUM	RandomTree	0.715	0.668
(6) stopwords,POS,stem	RandomizableFiltered	0.712	0.663
(7) stopwords,POS,stem, SYNSETS,word,NUM	RandomTree	0.714	0.663
(8) stem, lemma,NUM	RandomizableFiltered	0.709	0.662
(9) stopwords,POS,stem, word	RandomizableFiltered	0.699	0.662
(10) stopwords,POS,SYNSETS, lemma,word,NUM	RandomizableFiltered	0.700	0.661
(11) POS,SYNSETS, word,NUM	RandomizableFiltered	0.706	0.660
(12) POS,stem,NUM	RandomTree	0.704	0.655
(13) stopwords, stem	RandomizableFiltered	0.689	0.654
(14) POS,stem	RandomTree	0.700	0.650
(15) POS,stem, SYNSETS,NUM	RandomTree	0.685	0.643

As expected, the results with 2 classes significantly exceeded the results evaluated with 4 classes, achieving an improvement of approximately 7 points over the ROC Area in the best classifier. Furthermore, the same pattern is observed: KStar with the simplest features of the post along with SYNSETS and NUM, gave the best results. It should be noted that the results of algorithms using Decision Tree (RandomCommittee, RandomizableFiltered, and RandomTree) were placed in almost all the first positions, except for one case. In most of them, the POS features were present.

The Life Corpus used in the present study was created to be used as a basis in a suicide prevention system based on machine learning. This system (still under development) parts from on the detection of messages of possible suicidal ideas to send notices to then suicide prevention agencies. Therefore, it requires a balance between true positives and false positives of the risk class with true positives and false positives of the class that is not at risk. This is since the system must detect suicidal ideation messages as possible to reach as many people who need help but reduce false-positive notices and avoid overloading prevention services.

The balance relies a large extent on the capacity of these prevention agencies [45] and may vary according to available resources. Therefore, in unbalanced systems, measures such as the F-measure or ROC Area may not be the most adequate to detect which classifier is the best to use in each moment, for the suicide prevention services.

Maybe the features with the classifier that gives the best result detect too many False Positive (overloading the services) or detect a few cases of True Positive, when what the prevention service needs are messages from peoples that need help (and these messages do not arrive).

**Figure 1:**
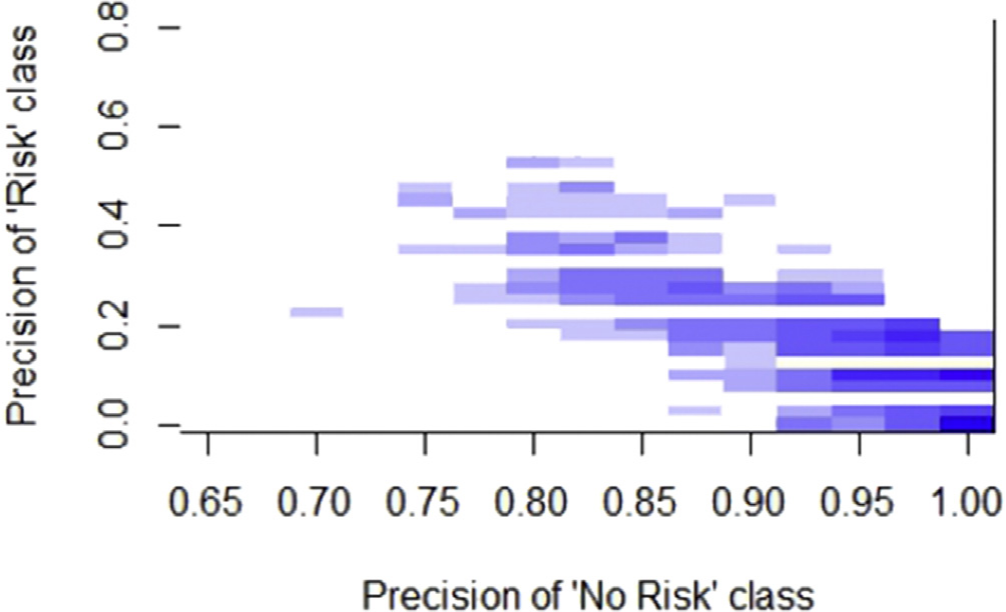
Representation of the Precision Bidimensional Index (PBI) of the results of the corpus with two classes.

For these reasons we decided to create a two-dimensional measure called the Precision Bidimensional Index (PBI) represented in the heat graph in Figure 1 where the Y-axis represents the proportion of True Positives with respect to the total of all samples classified as “Risk” (Equation 5) and on the X-axis the same proportion but with the class “No Risk” (Equation 6).

In this way, the points that most interest us are those located in the upper-right corner. A point located at the edge of this corner represents the perfectly classified, where both precision_risk_ and precision_no_risk_ for risk messages would be optimal. However, as seen in Figure 1, the results accumulate mostly in the lower right (those classifiers that are always classified as “No risk”) and go up to a point centered in 0.8.

This graph reflects, in the X-axis, the classifying the messages of no risk, which are not of interest for the prevention services and, in the Y-axis, the messages with the risk of suicidal ideation or depression, which would be the messages to send to such services. In this way, the most interesting points for these services will be those that are located on top of the graphic and that are more to the right because this will return more risk messages and few messages that do not indicate any danger.

Therefore, prevention services will always choose the system above because of the same number of false-positive messages, thus will have more true positives. But it will depend on the workload of the prevention services that will choose the column because if they have a lot of workloads, they will be interested in applying the system that makes the least mistakes in the “No Risk” even if some of the “Risk” escapes. The points located on top are listed in Table 6. In all these systems, the POS feature is relevant, being the only one that appears in all.

**Table 6:** The classifiers and features used for the learning models more relevant follow the PBI.

Corpus features	Classifier	F1	ROC Area
POS,SYNSETS, lemma,word	RandomCommitte	0.958	0.310
POS,stem	RandomTree	0.944	0.345
stopwords,POS,stem, SYNSETS,word,NUM	RandomTree	0.930	0.414
stopwords,POS,stem, SYNSETS,lemma, word,NUM	RandomizableFiltered	0.915	0.448
POS,SYNSETS,NUM	Kstar	0.901	0.448
POS,stem,NUM	RandomTree	0.901	0.414
POS,stem, SYNSETS,NUM	RandomTree	0.887	0.517
stopwords,POS,stem	RandomizableFiltered	0.887	0.448
POS,NUM	RandomTree	0.873	0.586
stopwords, stem	RandomizableFiltered	0.845	0.517
POS,SYNSETS, word,NUM	RandomizableFiltered	0.831	0.517
stem, lemma,NUM	RandomizableFiltered	0.831	0.586
stopwords,POS,SYNSETS,	RandomizableFiltered	0.817	0.517
stopwords,POS,stem,NUM	RandomizableFiltered	0.803	0.621
stopwords,POS,stem, word,lemma, word,NUM	RandomizableFiltered	0.789	0.724

Also, the decision trees or variants are usually in these systems. The point, in which the system obtains the highest accuracy for the risk class is the one located at a non-risk accuracy of 0.799, but it is also one of the systems on the list that uses most features. This shows that, although the POS is the characteristic that best discriminates the types of messages, it adds features that help the classification accuracy of the `` Risk '' class, although the `` No-Risk '' category gets worse.

## 5 Discussion

The machine learning algorithms that have obtained the best results are KStar and RandomCommittee when they have been used with features with more generalization capacity, such as the Part-Of-Speech (POS), the semantic identifier of the term (SYNSET) and the substitution of numbers by a unique identifier (NUM). The characteristics such as the root of the words (STEM), the lemma (LEMMA) and the word bag (WORD) have been relegated to lower positions, probably because the corpus is still too small for these features to appear in both the training and test samples.

It should be noted that eliminating common words (stopwords) such as prepositions, determinants, articles, certain verbs, etc., tends to give worse results, probably because some of the excluded terms (such as `no ',` a lot', ' be ', ...), in combination with other characteristics, are relevant to assess the risk of suicidal ideation and, therefore, it is not advisable to discard them.

On the other hand, during this work, we have determined realized that the usual measures to assess the quality of results in unbalanced systems (F-measure, ROC Area) not be the most adequate for suicide prevention, since in these circumstances, the classifier that provides the best result, can detect, due to imbalance, many false positives (overloading the services) or detect some cases of true positive, when what the prevention service needs are messages from people who need help, according to the needs of each prevention center or according to their message volume.

That is why we propose a new measure called Precision and Recall Bidimensional Index (PBI) that shows us, in a two-dimensional graph, the precision of classifying a message as Risk with respect to the coverage of risk messages, in such a way that systems that appear in the highest part and more to the right of the figure, will be, generally, the most interesting, being able to increase the number of messages with suicidal ideation risk, that the centers receive at the cost of receiving more messages that do not have any relation to suicide or vice versa, depending on the workload at a given moment of these services.

This implies that choosing the system with the highest value of F-measure or ROC Area will not always be the most suitable. What is clear is that those systems on the top surface of the figure should always be chosen, being worse systems those that are below another system in the same column.

## 6 Limitation

A limitation in this research is that, because it is oriented to computer science, we do not delve into the area of psychology, which is addressed in other research related to the Life research platform, whose objective is the automatic detection of suicidal ideas, incitement to suicide or symptoms of depression, from texts written on social networks [27], and with the future goal of developing a support tool for well-trained doctors or specialists [4, 5, 39] to support the prevention of possible suicides.

## 7 Conclusions and future work

The world population, as of January 2019, is more than 7.676 million people, of which 800.000 people die by suicide each year, due to which the WHO recognizes suicide as a global public health priority. In the report, “Suicidal prevention: A global imperative”, in the Mental Health Action Plan 2013–2020, published in 2014 [50], the member states of the WHO have committed to reduce national rates of suicide by 10% before the year 2020. For this they agreed to elaborate and put into practice comprehensive national strategies of suicide prevention, to improve their information systems, update their scientific data, and perform collaborative research with universities on mental health.

In this framework, researchers have collected messages with suicidal ideas from social networks such as: Twitter [21, 29, 31, 41, 53]; Weibo [19, 54]; Netlog [10]; and other microblogs [17], which represent a low percentage of data, compared to the high daily traffic of information that occurs on social networks such as Facebook (4 new petabyte data) and Twitter (500 million Tweets) [22], but the information they provide is essential in trying to decrease the standardized annual global suicide rate from 11.4 per 100,000 population (883,500 suicides in2020) [50].

We focused on the evaluation of 28 supervised classifier algorithms with the default Weka parameters. Each classifier was assessed by 124 combinations of features extracted from the Life Corpus. The classifier that showed better performance was KStar using POS-SYNSET-NUM and POS-NUM features. We suppose that this is due to the small corpus size because these features generalize more than others and can include in the correct class more samples but with similar lexical or semantic components. However, features such as a bag of words, stems, or lemmas are less likely to see samples with similar terms.

Despite the small size of the corpus, we have obtained good results which are statistically significant, reaching 0.75 of F-measure and 0.81 of ROC Area with 2 classes and 0.72 of F-measure and 0.75 of ROC Area with 4 classes. All these results are statistically significative with a confidence margin of 95%. Both results use the Life corpus with a moderate Cohen's Kappa micro mutual agreement of 0.52. Although the corpus has a low mutual agreement, this was measured to compare the 4 classes and was not measured for the separation of Risk and No Risk. Therefore, it is to be assumed that this corpus will have a greater agreement if it had been measured with only two classes. However, the results are very similar, and these do not vary a lot, neither with the agreement difference nor the F-measure and ROC Area values.

The results of this evaluation and its statistical analysis demonstrate that the Life Corpus and some machine learning techniques could be suitable for detecting suicide ideation messages despite its small size. To assess the reliability of this corpus, the observed agreement as well as Cohen's Kappa statistic [7] were accompanied by Confidence Intervals (CI) to provide a more detailed reliability description. CIs give more information indicating a range of values (interval) that is likely to contain the true value, with a probability or confidence level. In our case, 95% Confidence Intervals and a significance level of α = .05 has been set. To verify whether our agreement is statistically significant (i.e. K ≥ 0), the Confidence Intervals provided should not include 0. The formula employed to obtain Confidence Intervals is described in [28].

Another advantage of this work, compared to others, is the use of a bilingual corpus (English and Spanish), which does not focus on a single social network, and it is freely available under a Creative Commons license. Moreover, unlike other works, the Life Corpus used in this investigation is specifically oriented to the detection of suicidal ideation and depression. However, most of the related corpus presented in Section 2 try to detect other indirect features, like emotions or feelings.

Of importance is the fact that, our results provide evidence that, within unbalanced systems, the usual measures to assess the quality of results (F-measure, ROC Area) may not be the most adequate for suicide prevention, because there can be many false positives or true positive, when what the prevention service needs are messages from people who need help.

We have shown that, generally a new measure called the Two-dimensional Accuracy and Recovery Index (GDP), in the case of unbalanced systems, can provide better results than the usual measures to assess the quality of the results (measure F, ROC area), which increases the number of messages with risk of suicidal ideation that the centers receive at the cost of receiving more messages that have no relation to suicide or vice versa.

In future work, we propose to create mechanisms to increase the size of the corpus without harming its quality through semi-supervised algorithms. The evaluation process of all these supervised systems has been quite costly computationally, which is why we want to include metaheuristics to select, in a more efficient way, the best characteristics in future more advanced models, as well as to choose the best properties of the classifiers. In this research, we have used the classification algorithms that come by default in Weka, but in future works the metrics of the classifiers will be modified, to have elements of comparison of our results, in addition to being able to make comparison with other works.

We would also like to obtain the Cohen's Kappa from the corpus with only two characteristics to assess the quality improvement of the corpus. Other minor tasks that we want to explore include detecting which common words are the ones that should be included, and which are not in this task or explore the possibility of using n-grams or, even, skip-grams.

Likewise, we foresee experiments with genetic algorithms to improve the performance of the selection of features, so that we can also track the parameters of each classifier. In this research, we used brute force to run 3472 different experiments, this was possible because the corpus was small. However, there are plans to expand the corpus considerably with social network data ethically [12, 47], and experiment with all possible combinations, which is a complex task given the computational costs with current technologies.

## Declarations

### Author contribution statement

R. Acuña, J. Gómez: Conceived and designed the experiments; Performed the experiments; Analyzed and interpreted the data; Wrote the paper.

A. Melgar: Analyzed and interpreted the data; Contributed reagents, materials, analysis tools or data; Wrote the paper.

### Funding statement

This research work has been partially funded by the University of Alicante (Spain), Generalitat Valenciana and the Spanish Government through the projects “Tecnologías del Lenguaje Humano para una Sociedad Inclusiva, Igualitaria y Accesible” (PROMETEU/2018/089), “Modelado del Comportamiento de Entidades Digitales Mediante Tecnologías del Lenguaje Humano” (RTI2018-094653-B-C22) and “INTEGER: Intelligent Text Generation, Generación Inteligente de Textos” (RTI2018-094649-B-I00).

### Competing interest statement

The authors declare no conflict of interest.

### Additional information

No additional information is available for this paper.
